# Navigating neurological disorders: harnessing the power of natural compounds for innovative therapeutic breakthroughs

**DOI:** 10.17179/excli2024-7051

**Published:** 2024-04-23

**Authors:** Rapuru Rushendran, Rukaiah Fatma Begum, Ankul Singh S, Pavithra Lakshmi Narayanan, Chitra Vellapandian, Bhupendra G. Prajapati, Pijush Kumar Paul

**Affiliations:** 1 Department of Pharmacology, SRM College of Pharmacy, SRM Institute of Science and Technology, Kattankulathur- 603 203, Tamil Nadu, India; 2 Department of Pharmaceutical Chemistry, SRM College of Pharmacy, SRM Institute of Science and Technology, Kattankulathur- 603 203, Tamil Nadu, India; 3 Shree S. K. Patel College of Pharmaceutical Education and Research, Ganpat University, Kherva, 384012, Gujarat, India; 4 Department of Pharmacy, Gono Bishwabidyalay University, Mirzanagar, Savar, Dhaka-1344, Bangladesh

**Keywords:** neuroprotection, natural compounds, animal sources, marine sources, neurological disorders

## Abstract

Novel treatments are needed as neurological issues become more frequent worldwide. According to the report, plants, oceans, microorganisms, and animals contain interesting drug discovery compounds. Alzheimer's, Parkinson's, and stroke reviews emphasize neurological disorders' complexity and natural substances' safety. Learn about marine-derived and herbal substances' neuroprotective characteristics and applications. Molecular pathways show these substances' neurological healing effects. This article discusses clinical usage of Bryostatin-1, Fucoidan, Icariin, Salvianolic acid, Curcumin, Resveratrol, etc. Their potential benefits for asthma and Alzheimer's disease are complex. Although limited, the study promotes rigorous scientific research and collaboration between traditional and alternative medical practitioners. Unexplored natural compounds, quality control, well-structured clinical trials, and interdisciplinary collaboration should guide future study. Developing and employing natural chemicals to treat neurological illnesses requires ethical sourcing, sustainability, and public awareness. This detailed analysis covers natural chemicals' current state, challenges, and opportunities in neurological disorder treatment.

See also the graphical abstract(Fig. 1).

## Introduction

Neurological disorders constitute a broad and complex category of medical conditions that afflict millions of individuals worldwide, transcending age, gender, and geography. These disorders, which encompass a multitude of ailments affecting the central and peripheral nervous systems, carry profound implications for both the individuals' living with them and whole society (Patel et al., 2016[114]). Characterized by a wide range of symptoms, neurological disorders can manifest as cognitive, motor, sensory, or autonomic dysfunction, and their impacts can extend well beyond physical and mental health. Neurological disorders have a profound global impact, affecting hundreds of millions of individuals. Each year, over 6 million lives are lost to strokes, with more than 80 % of these fatalities occurring in low- and middle-income countries. Worldwide, more than 50 million people grapple with epilepsy. Dementia afflicts approximately 47.5 million people globally, with an additional 7.7 million new cases arising annually. Alzheimer's disease stands as the predominant cause of dementia, contributing to 60-70 % of these cases (Hussain et al., 2023[57]). Migraine is prevalent in over 10 % of the world's population. As the global population ages, the prevalence of neurological disorders is on the rise, adding urgency to the need for innovative treatments and interventions that can alleviate suffering, enhance the quality of life, and reduce the economic burden associated with these conditions. The prevalence of neurological disorders varies significantly depending on the specific disorder, geographic region, and demographic factors. Neurological disorders encompass various conditions affecting the nervous system, including the brain, spinal cord, and peripheral nerves. Tension-type headaches and migraines are among the most prevalent neurological disorders worldwide. Migraines alone affect more than 1 billion people globally, making them the third most prevalent illness globally. Epilepsy is one of the most common severe neurological disorders, with an estimated 50 million people affected worldwide (Rushendran et al., 2023[123]). Alzheimer's disease, a progressive neurodegenerative disorder, affects over 50 million people globally. This number is expected to rise significantly as the population ages. Parkinson's disease is estimated to affect more than 6 million people worldwide. Its prevalence increases with age. Multiple sclerosis affects approximately 2.8 million people globally (Singh et al., 2022[132], 2023[133]; Suresh et al., 2022[136]). Its prevalence varies by region, with higher rates in North America and Europe.

The intricate and bidirectional connection between depression, anxiety, and neurological disorders is characterized by a complexity that extends beyond the conventional boundaries of mental health (Krishnan and Nestler, 2008[78]; Maj et al., 2020[91]). Although depression and anxiety are typically classified as mental health disorders, there is compelling evidence supporting a substantial interplay with neurological elements. Shared characteristics include imbalances in neurotransmitters, changes in the structure and function of brain, and involvement of inflammatory processes and immune system dysregulation (Giannakopoulou et al., 2021[47]; Remes et al., 2021[120]). The confluence of chronic stress, common risk factors, and genetic susceptibilities further contributes to the overlapping nature of these conditions. Additionally, medications prescribed for neurological disorders may exert an impact on mood. Managing chronic health conditions, particularly neurological disorders, poses challenges that can exacerbate or play a role in the onset of depression and anxiety (Mariotti, 2015[93]; McEwen, 2017[96]). Recognizing this intricate connection emphasizes the need for a comprehensive and holistic approach to comprehend and address health's mental and neurological facets. This underscores the crucial role of healthcare professionals in conducting thorough assessments to ensure accurate diagnosis and effective treatment.

Stroke stands as a major contributor to global mortality and disability, with millions of new cases reported annually. The prevalence varies by region and is influenced by lifestyle and risk factors. Amyotrophic lateral sclerosis is a neurodegenerative condition primarily impacting the motor system, yet it is increasingly acknowledged for its additional non-motor manifestations. Progressive muscle weakness and atrophy result from the loss of both upper and lower motor neurons in the motor cortex, brain stem nuclei, and the anterior horn of the spinal cord. While ALS typically begins focally, it subsequently extends to various body regions, and respiratory muscle failure commonly limits survival to 2-5 years post-onset. Extra-motor manifestations, observed in up to 50 % of cases, encompass alterations in executive dysfunction, behavior, and language difficulties. Notably, 10-15 % of patients exhibit such pronounced issues that they meet the clinical criteria for frontotemporal dementia (Masrori and Van Damme, 2020[94]). Autism is a genetic, developmental neurological disorder, and its prevalence varies by region and diagnostic criteria. In some areas, it affects as many as 1 in 54 children. Peripheral neuropathy, a condition affecting the nerves outside the central nervous system, has a broad range of causes (Masrori and Van Damme, 2020[94]). Its prevalence depends on the underlying condition but can be substantial. It's important to note that these statistics provide a general overview of the prevalence of common neurological disorders. The actual numbers may vary over time and across different populations. Additionally, as the global population ages and diagnostic capabilities improve, the prevalence of many neurological disorders is expected to increase, making them a significant public health concern (Pan et al., 2021[110]; Vaquerizo-Serrano et al., 2021[147]; Wang et al., 2023[153]). In this article, we delve into the world of neurological disorders, exploring their prevalence, challenges, and the pressing need for novel treatments, with a particular focus on the potential of natural compounds in revolutionizing neurological disorder drug discovery. Neurological disorders represent a critical public health challenge, given their prevalence, impact, and the limitations of existing treatments. Novel approaches, such as exploring the potential of natural compounds, offer hope for addressing these disorders by providing innovative, safe, and effective treatments to improve the quality of life for those affected.

## Unmet Needs and the Potential of NaturalCompounds for Neurological Disorders

**Figure 2:**
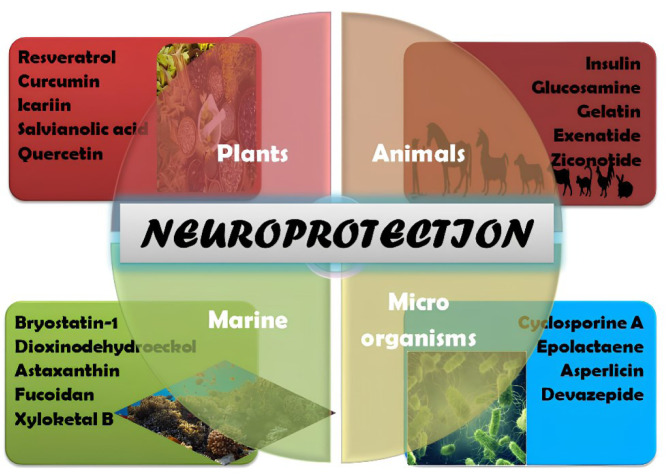
Natural compounds from diverse origins for neurological disorders

Neurological disorders encompass various conditions affecting the nervous system, including the brain, spinal cord, and peripheral nerves. They are a significant global health concern due to their prevalence and profound impact on individuals, families, and society. Conditions such as Alzheimer's disease, Parkinson's disease, epilepsy, multiple sclerosis, and stroke affect millions of people worldwide and are significant causes of disability and mortality. Many neurological disorders remain without effective cures or even disease-modifying treatments. Existing therapies often focus on managing symptoms rather than addressing the underlying causes of the disorders. Patients and their caregivers face a significant burden in managing these chronic and debilitating conditions. Neurological disorders impose substantial economic and social burdens. Healthcare costs for treatment and long-term care are substantial. The cognitive and physical impairments associated with these disorders often limit individuals' ability to work and participate in daily life, resulting in reduced quality of life and social isolation. As the global population ages, the prevalence of neurological disorders is expected to rise significantly. This demographic shift adds urgency to the need for innovative treatments and interventions that can slow disease progression or alleviate symptoms. Traditional drug discovery and development processes for neurological disorders have proven challenging. Identifying safe and effective compounds, especially for complex conditions like Alzheimer's or Parkinson's, has been formidable. Natural compounds, derived from plants, animals, microorganisms, or marine organisms, have gained attention as potential sources for novel neurological disorder treatments. Frequently, these compounds boast a rich tradition in traditional medicine and may present distinctive bioactive characteristics. Natural compounds can provide a holistic approach to addressing neurological disorders, potentially targeting multiple aspects of the disease pathology. This approach aligns with the growing recognition that neurological disorders often have multifactorial origins, illustrated in Figure 2(Fig. 2). Many natural compounds are well-tolerated and have a favorable safety profile, making them attractive options for long-term use. Additionally, their renewable and sustainable sources align with the need for environmentally responsible drug development.

## Natural Compounds as Potential Sources for Drug Discovery

Natural compounds have long been recognized as valuable sources for drug discovery due to their diverse chemical structures and potential therapeutic properties. Natural compounds represent a valuable and fertile ground for drug discovery due to their chemical diversity, historical use, bioactivity, and potential for creating effective and safe pharmaceuticals. The exploration of natural compounds continues to be a promising strategy in the search for novel treatments across a wide range of diseases and conditions. Natural compounds are derived from various sources, including plants, microorganisms, marine organisms, etc. This diversity provides a vast library of chemical structures to explore for potential drug candidates. Natural compounds often have complex and unique chemical compositions, which can lead to novel therapeutic properties. Many natural compounds have been used in traditional medicine for centuries, providing rich empirical evidence regarding their safety and efficacy. This historical knowledge can guide modern drug development efforts. Natural compounds often have specific biological activities. For example, plants produce secondary metabolites as a defense mechanism against predators and environmental stressors. Some of these compounds exhibit biological activities that can be harnessed for therapeutic purposes. Natural compounds frequently serve as a starting point for the development of pharmaceutical drugs. Scientists often isolate and modify these compounds to enhance their efficacy, safety, and bioavailability, leading to the creation of new drug candidates. Some natural compounds are highly selective in their actions, which can benefit drug development. They can target specific molecular pathways or receptors involved in disease processes with minimal off-target effects. Natural compounds can be used as building blocks for combinatorial chemistry, enabling the creation of large libraries of potential drug candidates with varying structures and properties. In an era of increasing environmental awareness, the sustainable and renewable nature of natural compound sources aligns with the demand for more environmentally responsible drug development practices. Many natural compounds have a long history of human consumption, making them more likely to be well-tolerated and safe for pharmaceutical use. The pharmacokinetics (absorption, distribution, metabolism, excretion) and pharmacodynamics (effect on the body) of some natural compounds are well-understood, which can expedite the drug development process. Some natural compounds work synergetic with other compounds, potentially enhancing their therapeutic effects while reducing side effects. The complexity and diversity of natural compounds can help address the issue of drug resistance, particularly in infectious diseases, by offering multiple mechanisms of action. The study of natural compounds has the potential to rejuvenate drug discovery by providing new avenues for drug development, especially in areas where traditional approaches have had limited success.

## Explore Emerging Trends in the Use of Natural Compounds for Neurological Disorder Treatments

At the time of our last knowledge update in November 2023, significant progress and notable developments had occurred in utilizing natural compounds to treat neurological disorders. It's important to acknowledge that subsequent developments may have transpired. Marine-derived compounds display diverse activities encompassing anti-inflammatory, anti-apoptotic, anti-oxidant, anticancer, and neuroprotective effects. Promisingly, certain compounds demonstrate potential in addressing neurological disorders like Alzheimer's disease, Parkinson's disease, stroke, and traumatic brain injury. The mechanisms of action often involve specific processes such as inhibiting protein aggregation, modulating oxidative stress, and regulating pathways associated with neuroinflammation. These compounds hail from various marine organisms, including bryozoans, sea cucumbers, mollusks, and sponges, underscoring the rich biodiversity of marine ecosystems. Herbal compounds sourced from Punica granatum, Cannabis sativa, and Centella asiatica showcase neuroprotective effects. Many of these compounds act as anti-oxidants, providing a defense against oxidative stress, a prevalent factor in neurodegenerative disorders. The compounds originate from a diverse array of herbs, emphasizing the potential inherent in natural sources for neuroprotection. Compounds sourced from animals exhibit neuroprotective effects (Table 1(Tab. 1); References in Table 1: Acosta et al., 2009[5]; Aksoy et al., 2017[8]; Bozorgi et al., 2020[21]; Duarte et al., 2005[38]; Haque et al., 2022[54]; Hwang et al., 2010[60]; Jamialahmadi et al., 2013[61]; Jhelum et al., 2022[62]; Joachim et al., 2014[65]; Kang et al., 2007[69]; Kaur et al., 2013[70]; Kim et al., 2013[73]; Li et al., 2016[84]; Nasr et al., 2019[104]; Ramalingam and Kim, 2016[118]; Shin et al., 2013[128]; Wang et al., 2022[151]; Yu et al., 2015[166]; Zamani et al., 2020[168]; Zhao and Brinton, 2006[174]) through diverse mechanisms, including the regulation of signaling pathways, the inhibition of inflammation, and the enhancement of neuronal survival. Experimental studies commonly employ animal models such as rats and mice to assess the neuroprotective potential of these compounds. Notably, several compounds in this category focus on alleviating neuroinflammation, a shared contributor to various neurological conditions. A recurring theme across all tables is the focus on compounds with neuroprotective properties, indicating a shared interest in developing therapies for protecting and preserving neuronal function. Compounds are derived from various sources, including marine organisms, herbs, and animal tissues, showcasing the exploration of biodiversity for potential neurological treatments. The compounds often target multiple pathways, suggesting a multifaceted approach to addressing neurological disorders by modulating inflammation, oxidative stress, and protein aggregation. Several compounds emerge as potential candidates for further research and development due to their efficacy in preclinical studies. The varied and optimistic terrain of natural compounds has been a beacon of hope in the exploration of new therapeutic possibilities for neurological disorders.

## Molecular Pathways of Natural Compounds in Neurological Disorders

**Figure 3:**
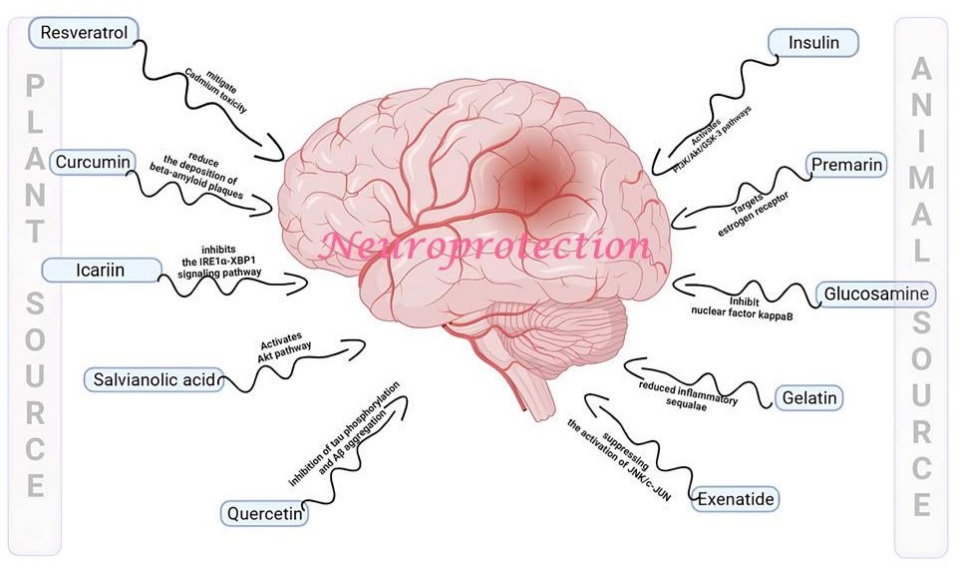
Illustration depicting the diverse pathways explored by neuroprotective agents derived from both plant and animal sources. Insulin activates PI3K pathway; Premarin targets estrogen receptors; Glucosamine inhibits NFκB pathway; Gelatin reduces inflammatory sequelae; Exenatide suppresses JNK activation; Quercitin inhibits tau phosphorylation and amyloid beta aggregation; Salvianolic acid activates AKT pathway; Icariin inhibits IRE1α-XBP1 pathway; Curcumin reduces deposition of beta-amyloid plaques; Resveratrol mitigates cadmium toxicity which collectively is involved in neuroprotection.

**Figure 4:**
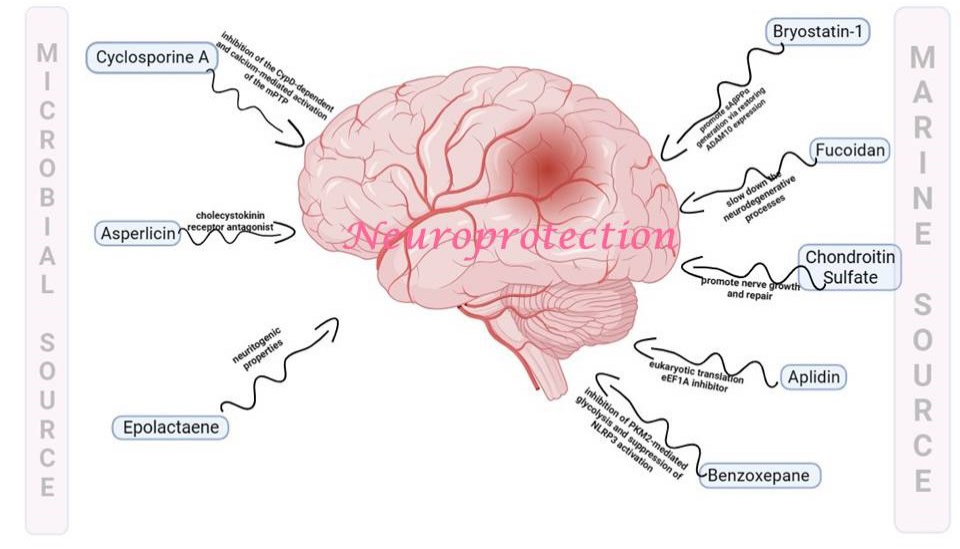
Schematic representation of the diverse pathways explored by neuroprotective agents derived from microbial and marine sources. Bryostatin 1 restores ADAM10 expression; Fucoidan slows down the neurodegenerative process; Chondroitin sulfate promotes nerve growth and repair; Aplidin acts as EF1A inhibitor; Benzoxepane inhibits PKM2N and suppresses the activation of NLRP3; Epolactaene acts as neuritogenic; Asperlicin acts as cholecystokinin antagonist; Cyclosporine A inhibits CYPD-dependent and activation of mPTP which collectively leads to neuroprotection.

**Figure 5:**
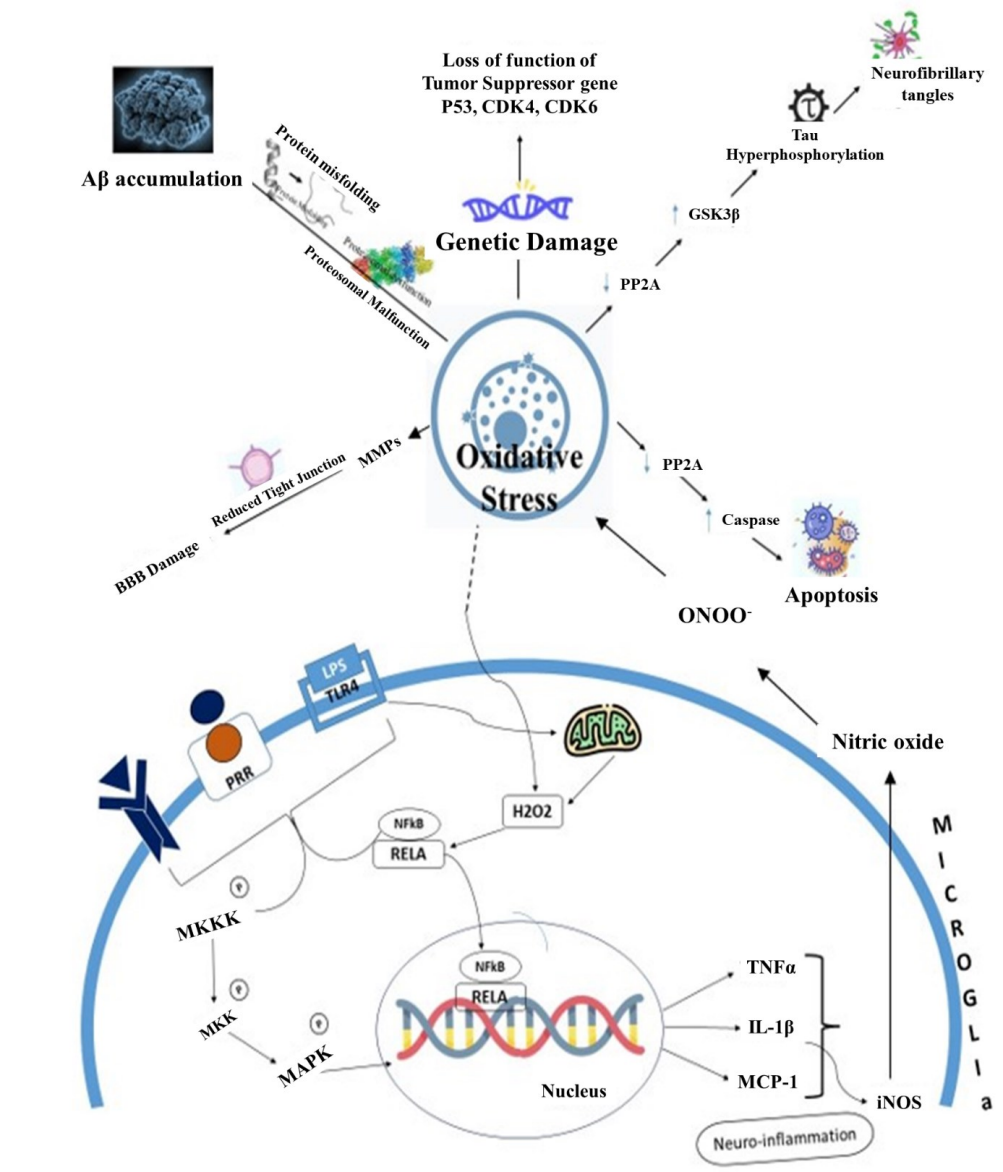
Pathways involved in oxidative stress-mediated neurological health impairment

Natural compounds often work at a molecular level to address neurological disorders by influencing various biological pathways and processes within the nervous system. Some natural compounds exhibit neuroprotective properties by shielding nerve cells from damage and promoting survival. This process can be crucial in conditions such as neurodegenerative diseases where neurons are progressively lost. Chronic neuroinflammation is a common feature of many neurological disorders. Natural compounds like curcumin and resveratrol have anti-inflammatory properties and can reduce inflammation in the nervous system. Research suggests that curcumin and resveratrol may help reduce inflammation in the nervous system by targeting specific inflammatory markers and pathways. For example, they may inhibit the activity of nuclear factor kappa B (Gonzales and Orlando, 2008[51]; Mazzanti and Di Giacomo, 2016[95]; Salehi et al., 2018[125]). Oxidative stress plays a role in various neurological disorders. Oxidative stress is intricately linked to the pathogenesis of numerous neurological disorders, playing a pivotal role in the progression of these conditions. The heightened metabolic activity of neurons, coupled with their relatively low anti-oxidant capacity, renders them particularly susceptible to the damaging effects of reactive oxygen species (Kim et al., 2015[74]; Li et al., 2015[86]; Pizzino et al., 2017[117]). This imbalance leads to cellular damage, including lipid peroxidation, protein misfolding, and DNA modifications, contributing to the dysfunction and demise of neurons. Mitochondrial dysfunction, another consequence of oxidative stress, not only disrupts cellular energy production but also amplifies ROS generation, creating a self-reinforcing cycle. Moreover, oxidative stress is intimately associated with inflammatory processes in the central nervous system, further accelerating neuronal injury and impairing overall neurological function. Neurodegenerative disorders, such as Alzheimer's disease, Parkinson's disease, and amyotrophic lateral sclerosis (ALS), showcase the cumulative impact of oxidative stress, where chronic exposure to ROS contributes to the aggregation of abnormal proteins and the progressive loss of neu-rons. Recognizing the central role of oxidative stress provides a foundation for exploring therapeutic strategies aimed at mitigating its effects and preserving neurological health (Singh et al., 2019[131]; Uttara et al., 2009[146]; Zhang et al., 2021[170]). Several pathways are involved in the impact of oxidative stress on neurological health. For instance, genetic damage can impair the function of tumor suppressor genes like P53, CDK4, and CDK6 (Fan et al., 2023[41]). Alternatively, oxidative stress may reduce the activity of PP2A while increasing GSK3β, leading to Tau hyperphosphorylation (Bartolome et al., 2022[17]). It can also promote matrix metalloproteinase (MMP) activity, which damages the blood-brain barrier (Hu et al., 2022[58]). Furthermore, oxidative stress can disrupt proteasomal function and cause protein misfolding, leading to the accumulation of amyloid beta proteins (Lévy et al., 2019[83]). Oxidative stress can activate the caspase pathway by reducing ATP levels, resulting in apoptosis (Zhuang et al., 2020[177]). Additionally, it can enhance the NFκB pathway, leading to the production of inflammatory molecules such as TNF alpha, IL1 beta, and MCP-1, which further stimulate the inducible nitric oxide synthase (iNOS) to produce nitric oxide, contributing to neuroinflammation (Chen et al., 2023[26]). Other factors like interferon-gamma (INF gamma), damage-associated molecular patterns (DAMPs), and lipopolysaccharides (LPS) can also activate the MAPK and NFκB pathways, exacerbating neuroinflammation (Zhang et al., 2021[172]). Natural compounds with anti-oxidant properties, such as vitamin E, vitamin C, and flavonoids, can neutralize harmful free radicals and reduce oxidative damage to nerve cells. Some natural compounds can influence the production, release, or function of neurotransmitters, the chemical messengers that transmit signals in the brain. This can help regulate mood, cognition, and other neurological functions. Figure 5 illustrates the pathways involved in oxidative stress-mediated neurological health impairment. Natural compounds like ginkgo biloba can improve blood circulation in the brain, which may benefit conditions associated with reduced cerebral blood flow, such as vascular dementia (Arulselvan et al., 2016[14]; Kurutas, 2016[81]; Teleanu et al., 2019[139]). Natural compounds facilitate neuroplasticity, the brain's ability to reorganize and adapt. This can be vital for recovery after brain injuries or for learning and memory. Some natural compounds can stimulate the production of neurotrophic factors, such as brain-derived neurotrophic factor. These factors promote the growth and maintenance of neurons. Figure 3(Fig. 3) provides a visual representation of the diverse pathways investigated by neuroprotective agents derived from both plant and animal sources and Figure 4(Fig. 4) provides a visual representation of the diverse pathways investigated by neuroprotective agents derived from both microbial and marine sources. This illustration highlights the complexity and versatility of these agents in promoting neuroprotection across various pathways. See also Figure 5(Fig. 5).

In Alzheimer's disease, the accumulation of beta-amyloid plaques is a hallmark. Natural compounds like curcumin have been investigated for their ability to reduce the formation and accumulation of these plaques (Mishra and Palanivelu, 2008[100]). Certain natural compounds can influence the activity of ion channels in nerve cells, affecting their excitability and signal transmission. Some natural compounds act as enzyme inhibitors, impacting the breakdown or production of specific molecules relevant to neurological disorders. For instance, acetylcholinesterase inhibitors are used to treat Alzheimer's disease (McGleenon et al., 1999[97]; Saxena and Dubey, 2019[126]; Subramanian et al., 2022[135]). Natural compounds can affect gene expression in neurons, influencing various processes related to neurological health and function. It's essential to note that the mechanisms of action can vary widely among different natural compounds, and their effectiveness may differ from one neurological disorder to another. Moreover, ongoing research continuously reveals new insights into how these compounds work at a molecular level, providing potential avenues for drug discovery and therapy development.

## Clinical Trials Conducted on Natural Compounds

As per the information available until our last update in November 2023, Bryostatin-1 had been studied in various preclinical investigations and clinical trials, primarily focusing on its potential therapeutic applications in neurological disorders and cancer. Bryostatin-1 has shown promise in preclinical studies for conditions such as Alzheimer's disease, multiple sclerosis, fragile X syndrome, stroke, traumatic brain injury, and depression. It demonstrated potential in addressing various neurological disorders. It is a potential inhibitor of protein aggregation, modulation of oxidative stress, and regulation of neuroinflammatory pathways. Bryostatin-1 possesses anti-inflammatory, anti-oxidant, MMPs inhibitory, and neurogenesis stimulatory properties, along with the added advantages of crossing the blood-brain barrier and being orally available, appears to be a suitable option for treating multiple sclerosis (Safaeinejad et al., 2018[124]). The impacts of bryostatin-1 likely encompass two aspects: acute safeguarding of the blood-brain barrier and chronic preservation of neuronal stability. Precision in timing and dosage is imperative to discern the most suitable protective treatment intervals. Modulation of protein kinase C presents a promising therapeutic avenue for averting the enduring consequences linked with neurotrauma (Lucke-Wold et al., 2015[90]). The neuro-pharmacological activity of other natural compounds derived from marine source were listed in Table 2(Tab. 2) (References in Table 2: Abdallah et al., 2020[1]; Ahmad et al., 2022[6]; Alves et al., 2018[9]; Ambati et al., 2014[10]; Anisha et al., 2022[13]; Cruz et al., 2009[30]; Delgado-Calle et al., 2019[32]; Di Cesare Mannelli et al., 2014[33]; Diao et al., 2023[34]; Egea et al., 2010[39]; Gao et al., 2020[44]; Gong et al., 2018[50]; Kim and Kong, 2010[76]; Miyata and Kitagawa, 2016[101]; Negi et al., 2017[105]; Oliveira et al., 2018[107]; Ortega and Cortés, 2012[108]; Ruiz-Torres et al., 2017[122]; Şimşek-Yavuz and Komsuoğlu Çelikyurt, 2021[130]; Tian et al., 2023[141]; Twede et al., 2009[145]; Wang et al., 2021[155], 2022[154]; Wyer et al., 2022[158]).

Fucoidan has demonstrated various therapeutic potentials, including anti-inflammatory, neuroprotective, anti-oxidant, and antitumor effects. Clinical trials or medical applications may be exploring its efficacy. The administration of oligo-fucoidan has the potential to decrease the lymphocyte proportion and lower inflammatory factor concentrations in individuals with asthma. This may contribute to the suppression of respiratory tract inflammation and an improvement in pulmonary function (Yeh et al., 2022[163]). A solitary administration of fucoidans can potentially impact the expression of genes associated with essential cellular processes. Furthermore, it substantiates earlier findings that fucoidans have an effect on immunity, cancer cells, inflammation, and neurological function (Gueven et al., 2020[53]). Additionally, the administration of fucoidan twice daily over a 90-day span did not significantly impact insulin resistance or other assessed parameters of cardiometabolic health in a group of obese, non-diabetic individuals. This lack of effect might stem from inherent inefficacy, adherence levels lower than recorded, or the possibility that a more extended therapy duration and higher baseline insulin resistance are necessary to achieve a notable impact (Wright et al., 2019[157]). Wheat peptides and fuconoids have brought fresh insights into dietary strategies for chronic gastritis. They have offered clinical and theoretical evidence supporting the development and commercialization of health foods tailored for this condition (Kan et al., 2020[68]). Fucoidan derived from Okinawa mozuku is deemed safe for consumption as a food product and has been shown to boost NK cell activity, particularly among males. Over a 12-week period, ingesting fucoidan at a dosage of 3 grams daily did not lead to troublesome adverse effects. Furthermore, no abnormalities were observed in blood or biochemical tests (Tomori et al., 2021[142]).

Icariin derived from Epimedium brevicornu Maxim, icariin has been investigated for its neuroprotective, anti-apoptotic, and anti-inflammatory effects and may be a subject of clinical interest. Icariin holds potential as an effective drug for treating patients with ankylosing spondylitis. However, additional follow-up studies involving larger group sizes are necessary to validate its efficacy (Wang et al., 2017[152]). Icariin and p-icariin exhibit anti-oxidant properties and provide hepatoprotection, as evidenced by decreases in serum liver injury markers and elevated levels of anti-oxidative enzymes. These alterations can potentially alleviate liver injury and are, to some extent, associated with the anti-oxidant properties of both compounds. While both components demonstrate similar free radical scavenging effects, p-icariin demonstrates superior hepatoprotective effects compared to icariin (Xiong et al., 2014[160]). There was a notable reduction in mortality, and 6-phosphate icariin exhibited a more pronounced effect than icariin. The likely mechanism behind the *in vitro* anti-viral activity of 6-phosphate icariin and icariin involves interference with virus replication and release. This suggests that the unique structure deserves further investigation for potential applications (Xiong et al., 2015[161]). Icariin exhibits various positive effects in the treatment of Alzheimer's disease. By combining pharmacological and molecular biological research, Icariin has the potential to be a promising candidate for accelerating the progress of traditional Chinese medicine in the clinical management of Alzheimer's disease (Zheng et al., 2023[175]). Icariin has the ability to control the expression of miR-144-3p and ATP1B2, while also promoting the phosphorylation of PI3K, Akt, and mTOR (Pan et al., 2022[109]). In conjunction with high-dose methylprednisolone, Icariin demonstrates synergistic effects in alleviating experimental autoimmune encephalomyelitis. This is achieved by modulating hypothalamic-pituitary-adrenal function and promoting anti-inflammatory and anti-apoptotic effects (Wei et al., 2016[156]).

Salvianolic acid obtained from Salvia miltiorrhiza has been studied for its neuroprotective and anti-ischemic properties and may be used in medical applications. Yimin has examining how food influences the pharmacokinetics of Salvianolic Acid A in healthy subjects, with the compound currently undergoing Phase I clinical trials (Yimin, 2019[164]). Salvianolic acid B demonstrates effective reversal of liver fibrosis in chronic hepatitis B. Compared to IFN-gamma, Salvianolic acid B exhibits superior results in reducing serum hyaluronic acid content, decreasing four serum fibrotic markers, and reducing ultrasound imaging scores. Salvianolic acid B is particularly suitable for anti-fibrotic treatment in cases of chronic hepatitis B with mild liver injury. Importantly, Salvianolic acid B shows no apparent side effects (Liu et al., 2002[89]). Additionally, Salvianolic acid B intralesional injections improved mouth opening and burning sensations in these oral submucous fibrosis patients (Jiang et al., 2013[64]). Salvianolic acid B exhibits neuroprotective effects against cerebral injury which was induced by ischemia or reperfusion (I/R) and holds promise as a valuable candidate for further advancement in clinical therapy development (Fan et al., 2018[42]). Salvianolic acid B has the capability to stimulate autophagy and facilitate the elimination of NLRP3, leading to neuroprotective and anti-depressant effects (Jiang et al., 2017[63]). It additionally mitigates neurological apoptosis in ischemic stroke by enhancing Stanniocalcin 1 (Bi et al., 2022[20]). The dose-dependent administration of Salvianolic acid B significantly inhibited the mRNA and protein overexpression of pro-inflammatory mediators, including ICAM-1, IL-1β, IL-6, IL-8, and MCP-1, in the penumbra cortex induced by ischemia/reperfusion (Xu et al., 2017[162]). Table 3(Tab. 3) (References in Table 3: Angeloni and Vauzour, 2023[12]; Balendra and Singh, 2021[15]; Begum et al., 2008[18]; Chang and So, 2008[23]; Che et al., 2017[25]; Chen et al., 2019[27]; Dai et al., 2021[31]; Dong et al., 2016[37]; Faridzadeh et al., 2022[43]; García-Villalba et al., 2023[45]; Ho et al., 2007[56]; Hosseini Adarmanabadi et al., 2023[57]; Khan et al., 2019[72]; Kim and Cho, 2021[77]; Kim et al., 2015[75]; Krishnapriya et al., 2022[79]; Laws and Smid, 2022[82]; Li et al., 2017[85]; Liu et al., 2022[88]; Majeed et al., 2022[92]; Park et al., 2012[111]; Pervin et al., 2018[115]; Shalini et al., 2021[127]; Tang et al., 2021[138]; Tongjaroenbuangam et al., 2011[143]; Tsai-Teng et al., 2016[144]; Zahiruddin et al., 2020[167]; Zhang et al., 2022[171]; Zhu et al., 2022[176]; Zhuang et al., 2012[178]) and Table 4(Tab. 4) (References in Table 4: Bermejo-Bescós et al., 2008[19]; Falsig et al., 2004[40]; Goetz et al., 1985[48]; Herranz, 2003[55]; Kakeya et al., 1995[66]; Kalantari-Dehaghi et al., 2013[67]; Kawakami et al., 2011[71]; Lima et al., 2017[87]; Murata, 2008[103]; Parkinson Study Group, 2004[112]; Zhang et al., 2015[169], 2019[173]) enumerate the neuro-pharmacological effects of additional natural compounds sourced from plant and microorganism origins.

Compounds from Cannabis sativa, especially phytocannabinoids, are reported to have potential neuroprotective effects. Cannabidiol (CBD) is a well-known cannabinoid that has been studied for its medicinal properties. Administration of oral medicinal cannabinoids may alleviate symptom burden in the palliative care of advanced cancer such as glioblastoma multiforme (Doherty and de Paula, 2021[35], Good et al., 2019[52]). CBD's can be utilized as a therapeutic potential in addressing neurological conditions such as Alzheimer's disease, Parkinson's disease, and epilepsy (Tambe et al., 2023[137]). Cannabidiol demonstrated a notable decrease in seizures associated with tuberous sclerosis complex when compared to a placebo. The safety profile of the 25 mg/kg/day dosage was superior to that of the 50 mg/kg/day dosage (Thiele et al., 2021[140]). Both transdermal cannabidiol doses (195 mg and 390 mg) were well tolerated and deemed safe in drug-resistant epilepsy adults. No significant difference in effectiveness was observed between cannabidiol and the placebo during the double-blind treatment phase. The open-label extension confirmed the enduring safety, tolerability, and acceptance of transdermal cannabidiol delivery (O'Brien et al., 2022[106]). Clinical reports suggest that cannabidiol may have the ability to reduce stress and anxiety (Spinella et al., 2021[134]). The safety and potential therapeutic use of hemp-derived cannabidiol is thus beneficial for alleviating pain associated with arthritis (Verrico et al., 2020[149]). The phytocannabinoid CBD exhibits anti-seizure and neuroprotective properties. Similar to endocannabinoids, CBD can modulate various aspects of neuronal function, including excitability, pain, inflammation, feeding regulation, learning and memory, and emotion regulation. Recent research indicates that CBD reduces inflammation, safeguards against neuronal loss, normalizes neurogenesis, and is an anti-oxidant. Cannabinoids exert diverse pharmacological effects through the activation of CB1 and CB2 receptors. While the psychoactive effects of THC are attributed to the activation of CB1, the mechanisms underlying the neuroprotective effects of CBD are still under investigation (Reddy, 2023[119]). CBD has been identified as generally safe and efficacious for treating seizures that resist conventional therapies in children experiencing severe early-onset epilepsy (Golub and Reddy, 2021[49]). CBD demonstrates anti-neuroinflammatory activity by suppressing NADPH oxidase-mediated reactive oxygen species, as well as downregulating the TLR4-NFκB and IFN-β-JAK-STAT pathways (Yousaf et al., 2022[165]). The findings from scientific studies conducted thus far on the clinical application of CBD could offer hope for patients who do not respond to conventional anti-epileptic medications (Silvestro et al., 2019[129]). To ascertain the effectiveness of CBD as a neuroprotective agent, extensive and well-designed randomized clinical trials will be required to obtain conclusive results regarding its potential as a therapeutic approach for diseases like Parkinson's and Alzheimer's (Viana et al., 2022[150]).

Resveratrol, present in grapes, berries, peanuts, red wine, and *Polygonum cuspidatum*, has garnered attention in medical research due to its studied neuroprotective effects. It is also considered promising in the treatment of colorectal cancer (CRC) by influencing crucial molecules and signaling pathways associated with cancer, including SIRT1, P53, P21, ROS, COX-2, AMPK, BMP7, Wnt, caspases, NO, NF-κB, TNFs, EMT, and the pentose phosphate pathway (Vernousfaderani et al., 2021[148]). Human clinical trials exhibit significant variations in the administered doses of resveratrol and the duration of treatment. In general, the notable impacts of resveratrol include a decrease in body weight among obese individuals and a partial decline in systolic blood pressure, fasting blood glucose, and HbA1c levels in some clinical trials involving patients with diabetes mellitus (Breuss et al., 2019[22]). The trajectory suggests that we are entering an era where approaches to treatments and strategies, especially nutritional interventions like resveratrol supplementation, aimed at addressing obesity and metabolic syndrome, will require a personalized approach tailored to each individual to maximize effectiveness (Chaplin et al., 2018[24]). Using nano-formulations of resveratrol might be the preferable strategy, considering their potential ability to target specific sites and minimize toxicity. It appears prudent to initiate new trials involving resveratrol nano-formulations or to further develop and refine previously validated innovative formulations. Considering the existing gaps, a substantial amount of work still needs to be undertaken before resveratrol can be regarded as a viable therapeutic agent for cancer treatment (Ren et al., 2021[121]). Resveratrol safeguards dopaminergic neurons from apoptosis, a hallmark of Parkinson's disease, by enhancing mitochondrial health through the upregulation of mitophagy and mitochondrial biogenesis (Kung et al., 2021[80]). Resveratrol hinders the activation of NF-κB and NLRP3 inflammasomes while diminishing the production of inflammatory cytokines. Its impact on reducing reactive oxygen species and oxidative stress is likely mediated through Nrf2 and its downstream anti-oxidant genes. The neuroprotective effects of resveratrol are impeded by the AMPK inhibitor (Chiang et al., 2022[29]).

Curcumin found in Curcuma longa has been extensively studied for its anti-inflammatory and neuroprotective properties. It is used in various medical applications (Zia et al., 2021[179]). Extensive research has been conducted on the neuroprotective effects of curcumin, with clinical trials aimed at substantiating these claims. However, the trials revealed that despite being a well-tolerated natural compound, curcumin did not demonstrate efficacy in improving the quality of life or clinical symptoms for patients with Parkinson's disease (Ghodsi et al., 2022[46]). Curcumin, a naturally occurring polyphenolic phytochemical renowned for its potent anti-inflammatory and anti-oxidant characteristics, in conjunction with IFN β-1a treatment, may boost the efficacy of IFN β-1a in managing radiological signs of inflammation in multiple sclerosis. However, despite a notable dropout rate, curcumin does not seem to provide neuroprotective effects, as indicated by clinical and MRI parameters (Petracca et al., 2021[116]). The nano-curcumin and coenzyme Q10 may collaborate to exert neuroprotective effects by modulating inflammation and oxidative stress. This suggests a potential synergistic impact of nano-curcumin and Co-Q10 on the clinical features of migraines (Parohan et al., 2021[113]). The examination of curcumin's efficacy as a supplementary agent alongside standard anti-psychotic medications in individuals with chronic schizophrenia uncovered that incorporating curcumin as an add-on to anti-psychotics for addressing negative symptoms could present a novel and safe therapeutic avenue in schizophrenia management. Nonetheless, it is crucial for these findings to be validated through additional studies (Miodownik et al., 2019[99]). Studies on both human subjects and experimental models of migraine have highlighted the involvement of COX-2/iNOS in the neuroinflammatory pathogenesis of migraines. Omega-3 fatty acids and curcumin, an active polyphenol found in turmeric, exhibit anti-inflammatory and neuroprotective effects by suppressing the expression of iNOS and COX-2 genes and their serum levels. These results suggest that a combination therapy involving ω-3 fatty acids and nano-curcumin holds promise as a novel and practical approach for preventing migraines (Abdolahi et al., 2019[2]). Nano-curcumin and ω-3 fatty acids have shown neuroprotective effects through modulation of IL-6 gene expression and CRP levels and can be considered as a promising target in migraine prevention. Indications suggest that tumor necrosis factor (TNF)-α contributes to the neuroimmune pathogenesis of migraines (Abdolahi et al., 2017[4], 2018[3]). The nano-curcumin appears to be a safe addition to treatment and may enhance the likelihood of survival in ALS patients, particularly those with pre-existing bulbar symptoms. However, to validate these observations, further studies with larger sample sizes and extended durations are essential (Ahmadi et al., 2018[7]). Research on curcumin has been explored due to its robust neuroprotective properties in mitigating damage resulting from spinal cord injury. Although the mechanism by which it preserves the function of the blood-spinal cord barrier remains unclear, the observed enhancement in motor function post-spinal cord injury raises intriguing possibilities for its potential role in improving the integrity of the blood-spinal cord barrier (Mokhber et al., 2014[102]). The research investigation of alterations in NF-κB DNA binding activity when subjected to TNF-α treatment both before and after intervention showed that the pre-intervention samples rose significantly in mean NF-κB DNA binding activity in response to TNF-α. Interestingly, there was an absence of NF-κB induction by TNF-α in the post-intervention samples. These results imply a potential protective function against human oxidative stress, achieved through administering a compound comprising four essential natural agents. Further exploration and research on this compound can potentially contribute to developing strategies aimed at shielding individuals from the adverse impacts of oxidative stress (Dominiak et al., 2010[36]).

Quercetin, a flavonoid compound, is abundant in various plant-based foods, including fruits, vegetables, leaves, and grains. Dietary sources rich in quercetin encompass apples, onions, berries, citrus fruits, red grapes, cherries, broccoli, leafy greens, tea, and red wine. Moreover, quercetin can be acquired through the use of dietary supplements (Anand David et al., 2016[11]). The association between quercetin and cognitive performance in Alzheimer's disease thus exerts its potential as a key compound in clinical applications (Khan et al., 2019[72]). Quercetin collaborates with agents to enhance therapeutic efficacy by modulating signal molecules and interrupting the cell cycle. Synergistic therapy allows for a reduction in agent doses, minimizing the risk of potential toxicity and side effects during treatment. While quercetin treatment may carry some possible side effects, it remains safe within anticipated usage conditions. Consequently, quercetin holds application value and promising potential as a clinical drug. Additionally, as the principal effective therapeutic component in traditional Chinese medicine, quercetin may be efficacious in treating and preventing (Zou et al., 2021[180]). Quercetin provides effective protection against seizure induced neuron death both *in vitro *and* in vivo* studies. It also mitigates impairment in cognitive function through modulation of the Nrf2/SIRT1/GPX4/SLC7A11 pathway (Xie et al., 2022[159]). Quercetin has demonstrated robust bioactivity in the fields of wound healing, neuroprotection, and anti-aging research (McKay et al., 2023[98]). High doses or extended administration of quercetin-conjugated superparamagnetic iron oxide nanoparticles can enhance cognitive function and stimulate neurogenesis without inducing toxicity. This can be attributed to QC's ability to impede protein aggregation and counteract iron overload through activities such as iron chelation, regulation of iron homeostasis genes, radical scavenging, and mitigation of the Fenton/Haber-Weiss reaction (Bardestani et al., 2021[16]). Quercetin regulates neurotransmitter levels, enhances the regeneration of hippocampal neurons, ameliorates hypothalamic-pituitary-adrenal (HPA) axis dysfunction, and diminishes inflammatory states and oxidative stress (Chen et al., 2022[28]).

## Highlights

- Numerous natural compounds exhibit excellent tolerability and boast a favorable safety profile, making them appealing choices for the prolonged management of chronic neurological conditions.
- Natural compounds frequently serve as starting points for developing pharmaceutical drugs. Isolation and modification enhance efficacy, safety, and bioavailability.
- Marine-derived compounds show promise in addressing neurological disorders, including Alzheimer's, Parkinson's, stroke, and traumatic brain injury. Mechanisms often involve inhibiting protein aggregation, modulating oxidative stress, and regulating neuroinflammatory pathways.
- Bryostatin-1 showed promising results in preclinical studies for Alzheimer's, multiple sclerosis, fragile X syndrome, stroke, traumatic brain injury, and depression.
- Fucoidan exhibits therapeutic potentials including anti-inflammatory, neuroprotective, anti-oxidant, and anti-tumor effects.
- Icariin shows potential effective treatment for ankylosing spondylitis and exhibits anti-oxidant properties, providing hepatoprotection.
- Salvianolic acid B is effective in reversing liver fibrosis in chronic hepatitis B and exhibits neuroprotective effects against cerebral injury induced by ischemia or reperfusion.
- CBD considered safe and efficacious for treating seizures in severe early-onset epilepsy.
- Resveratrol inhibits NFκB and NLRP3 inflammasomes, reducing inflammatory cytokines and oxidative stress.
- Quercetin collaborates with other agents for enhanced therapeutic efficacy.
- Nano-curcumin and coenzyme Q10 collaboration shows potential neuroprotective effects.

## Challenges and Limitations

The use of natural compounds from plants and herbs introduces variability, leading to inconsistent treatment outcomes. Scientific evidence supporting their efficacy, compared to pharmaceutical drugs, is often limited, necessitating further research for validation in neurological conditions. Challenges include potential interactions with medications, difficulty determining dosages, and limited bioavailability. Natural compounds may induce side effects, have slower therapeutic onset, and face varying regulatory oversight, potentially resulting in subpar products. Costly and limited availability can hinder access for neurological patients, and adherence challenges arise from taste, odor, and dosing requirements. Patients' beliefs and ethical/environmental concerns also impact their effectiveness. As they are not suitable for all neurological conditions, natural products may be limited to certain conditions, while conventional drugs are often the primary treatment option. 

## Future Directions

Unearthing novel natural compounds holds the key to innovative therapies for neurological disorders. This involves investigating uncharted territories like unexplored plant species, marine life, and microorganisms for bioactive substances. Standardizing and ensuring the quality of these compounds are paramount, requiring stringent testing methodologies for uniformity. Understanding their mechanisms of action is crucial for comprehending their interactions with neurological pathways. To enhance efficacy, efforts focus on improving bioavailability through advanced delivery methods and dedicated clinical trials. Personalized medicine is explored, considering individual responses based on genetic factors. Investigating synergies with conventional pharmaceuticals and examining potential interactions are avenues for improved therapeutic outcomes. Long-term studies are vital for assessing prolonged safety, addressing concerns associated with extended use. Regulatory measures and standardized guidelines ensure quality and safety, promoting ethical sourcing and sustainability. Collaboration among researchers, pharmacologists, chemists, and clinicians accelerates the translation of findings into effective treatments. Public awareness and education play a crucial role in ensuring safe and informed adoption of natural compounds. Addressing issues related to intellectual property, safeguarding traditional knowledge, and ensuring equitable access are of significant importance. Exploring the preventive potential of natural compounds and utilizing digital health technologies for real-world effectiveness are promising avenues for future research in neurological disorders.

## Summary and Conclusion

In conclusion, neurological disorders present a significant global health challenge, affecting millions of lives and posing complex medical, societal, and economic burdens. The prevalence of conditions such as Alzheimer's disease, Parkinson's disease, epilepsy, and migraines underscores the urgent need for innovative treatments. The impact of these disorders extends beyond physical and mental health, influencing the overall quality of life for individuals and their communities. The current landscape of neurological disorder treatments faces limitations, with many existing therapies focusing on symptom management rather than addressing underlying causes. Additionally, the aging global population contributes to the escalating prevalence of these disorders, emphasizing the necessity for novel interventions. Natural compounds, derived from diverse sources such as plants, marine, microorganisms, and animal, emerge as promising candidates for revolutionizing neurological disorder drug discovery. The multifaceted properties of natural compounds, including neuroprotection, anti-inflammation, and anti-oxidant effects, offer a holistic approach to address the complex nature of neurological disorders. Exploring the potential of marine-derived compounds, herbal compounds, and other natural sources unveils a rich diversity of bioactive substances with neuroprotective effects. These compounds hold promise for conditions like Alzheimer's, Parkinson's, stroke, and traumatic brain injury, offering novel avenues for therapeutic development. The molecular pathways through which natural compounds operate in neurological disorders, influencing inflammation, oxidative stress, neurotransmission, and gene expression, provide a comprehensive understanding of their mechanisms of action. These compounds exhibit potential in modulating various aspects of neurological health and function. While embracing natural compounds as potential treatments, it is crucial to recognize the challenges and limitations associated with their use. Standardization, evidence-based support, and addressing issues of quality control are essential for their integration into mainstream medical practice. Moreover, considerations such as potential interactions, dosage determination, and adherence must be carefully navigated. Looking ahead, future directions in natural compound research for neurological disorders involve ongoing exploration of uncharted compounds, enhancing quality control measures, understanding mechanisms of action, and conducting well-structured clinical trials. Embracing personalized medicine, investigating combinatory approaches, and ensuring ethical sourcing practices are pivotal for advancing the field. In this dynamic landscape, collaboration among researchers, pharmacologists, clinicians, and traditional medicine practitioners is essential for translating research findings into effective treatments. Public awareness and education play a crucial role in fostering safe and informed utilization of natural compounds.

## Notes

Chitra Vellapandian, Bhupendra G. Prajapati (Shree S. K. Patel College of Pharmaceutical Education and Research, Ganpat University, Kherva, 384012, Gujarat, India; E-mail: bhupendra.prajapati@ganpatuniversity.ac.in) and Pijush Kumar Paul (Department of Pharmacy, Gono Bishwabidyalay University, Mirzanagar, Savar, Dhaka-1344, Bangladesh; E-mail: pijushpaul.pharmacy@gonouniversity.edu.bd) contributed equally as corresponding author.

## Declaration

### Credit author statement 

RR, RFB, AS, PL: Writing - original draft, writing - review and editing. CV, BP, PP: Conceptualization, data curation, formal analysis, writing - review and editing. All the authors critically reviewed the manuscript for intellectual content. All authors approved the final version of the manuscript for publication.

### Conflict of interest

The authors declare that they have no conflict of interest. 

### Acknowledgment 

We express our sincere gratitude to all the supervisors and professors of SRM College of Pharmacy, SRM Institute of Science and Technology, Kattankulathur, Tamil Nadu, India who extended their contribution and support in this work. The author Rapuru Rushendran would like to sincerely thank “The Ministry of Tribal Affairs”, Government of India for providing the National Fellowship with award number 202122-NFST-AND-00924. This study was also supported via funding from Prince Sattam bin Abdulaziz University project number (PSAU/2023/R/1445). We also express our thanks to Ganpat University, for providing necessary support for this work.

## Figures and Tables

**Table 1:**
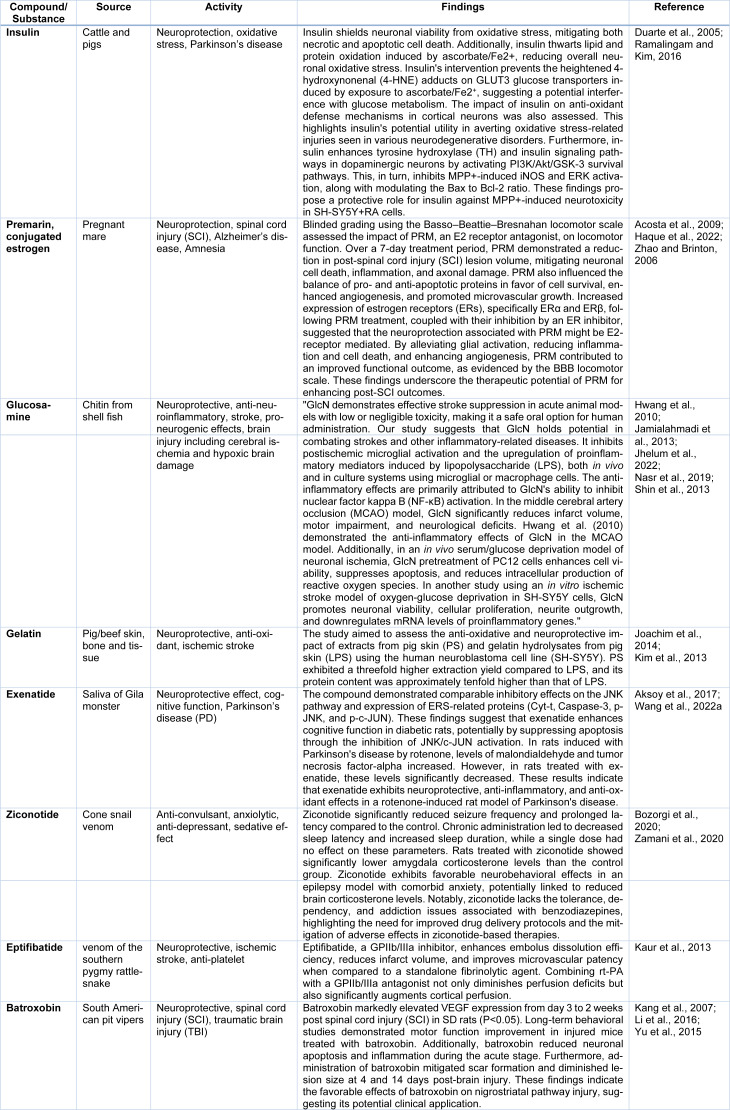
Compounds sourced from animals with potential neurological applications

**Table 2:**
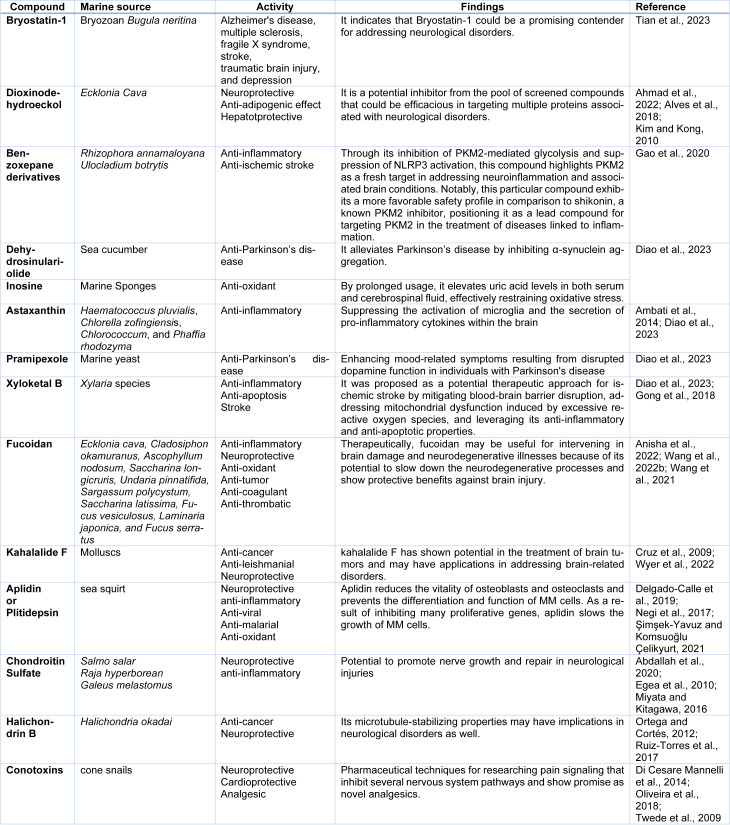
Marine derived compounds for neurological disorders

**Table 3:**
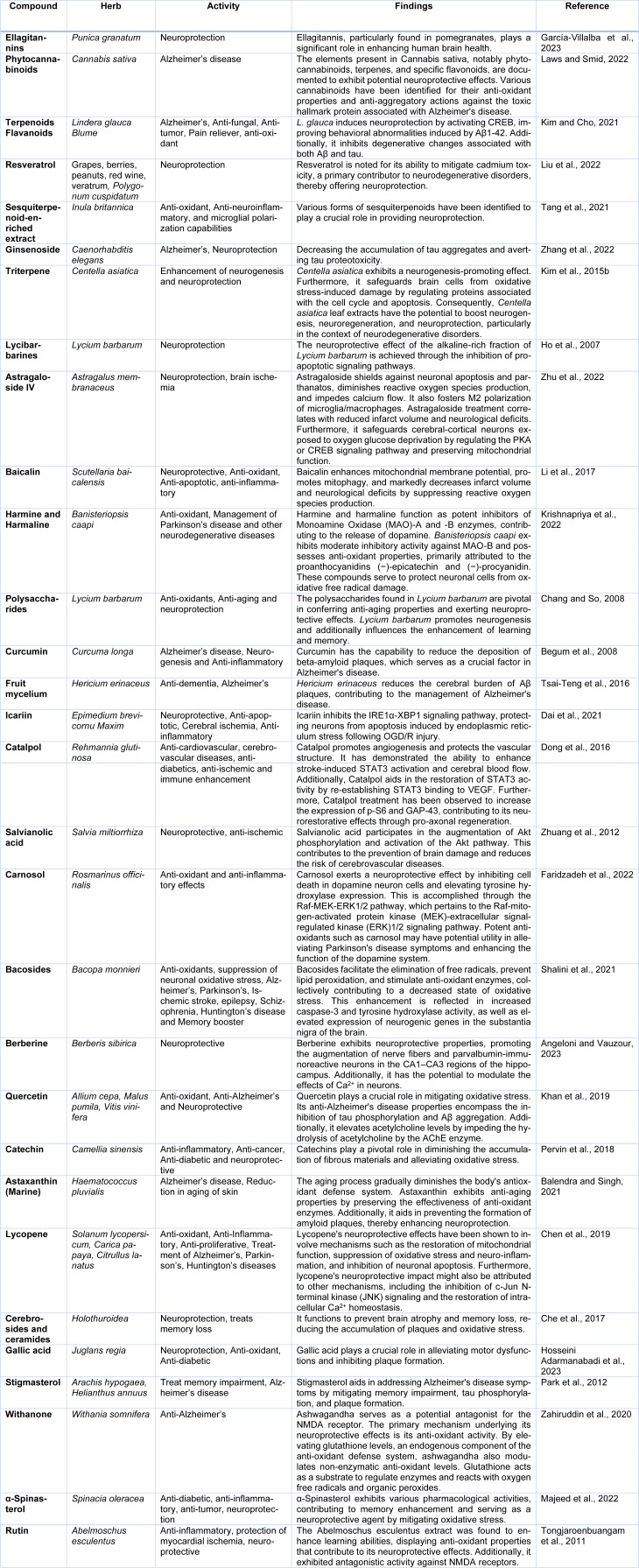
Herbal compounds and their neuroprotective activities

**Table 4:**
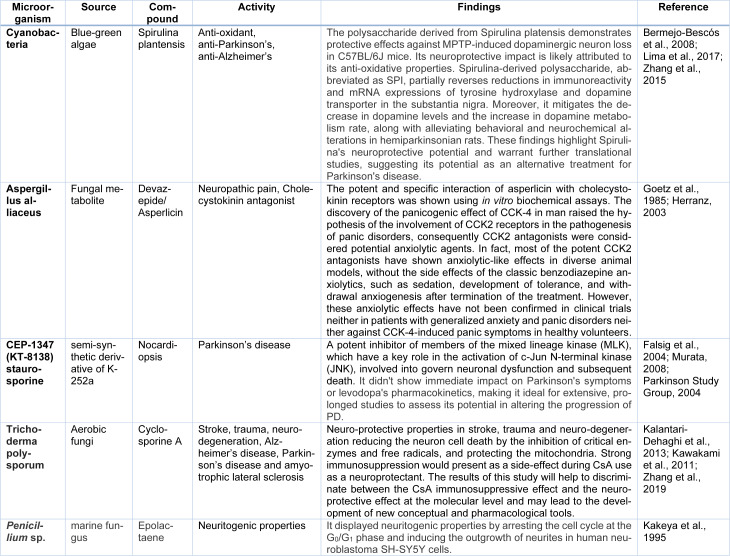
Neuroactive Compounds from Microbial Sources

**Figure 1:**
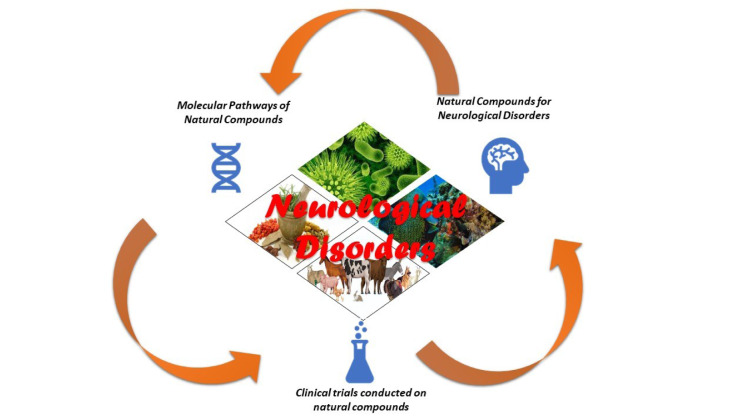
Graphical abstract
